# Integrating transcriptional, metabolomic, and physiological responses to drought stress and recovery in switchgrass (*Panicum virgatum* L.)

**DOI:** 10.1186/1471-2164-15-527

**Published:** 2014-06-26

**Authors:** Eli Meyer, Michael J Aspinwall, David B Lowry, Juan Diego Palacio-Mejía, Tierney L Logan, Philip A Fay, Thomas E Juenger

**Affiliations:** Department of Integrative Biology, Oregon State University, Cordley Hall 3029, Corvallis, OR 97331 USA; Department of Integrative Biology, University of Texas at Austin, 1 University Station C0930, Austin, TX 78712 USA; Hawkesbury Institute for the Environment, University of Western Sydney, Penrith, NSW 2751 Australia; USDA-ARS Grassland Soil and Water Research Laboratory, Temple, TX 76502 USA

**Keywords:** Drought, Recovery, Switchgrass, *Panicum virgatum*, Gene expression, RNA-seq

## Abstract

**Background:**

In light of the changes in precipitation and soil water availability expected with climate change, understanding the mechanisms underlying plant responses to water deficit is essential. Toward that end we have conducted an integrative analysis of responses to drought stress in the perennial C_4_ grass and biofuel crop, *Panicum virgatum* (switchgrass). Responses to soil drying and re-watering were measured at transcriptional, physiological, and metabolomic levels. To assess the interaction of soil moisture with diel light: dark cycles, we profiled gene expression in drought and control treatments under pre-dawn and mid-day conditions.

**Results:**

Soil drying resulted in reduced leaf water potential, gas exchange, and chlorophyll fluorescence along with differential expression of a large fraction of the transcriptome (37%). Many transcripts responded differently depending on time of day (e.g. up-regulation pre-dawn and down-regulation mid-day). Genes associated with C_4_ photosynthesis were down-regulated during drought, while C_4_ metabolic intermediates accumulated. Rapid changes in gene expression were observed during recovery from drought, along with increased water use efficiency and chlorophyll fluorescence.

**Conclusions:**

Our findings demonstrate that drought responsive gene expression depends strongly on time of day and that gene expression is extensively modified during the first few hours of drought recovery. Analysis of covariation in gene expression, metabolite abundance, and physiology among plants revealed non-linear relationships that suggest critical thresholds in drought stress responses. Future studies may benefit from evaluating these thresholds among diverse accessions of switchgrass and other C_4_ grasses.

**Electronic supplementary material:**

The online version of this article (doi:10.1186/1471-2164-15-527) contains supplementary material, which is available to authorized users.

## Background

Drought is the most important factor limiting ecosystem and agricultural productivity, and influencing plant community structure worldwide [1–6]. The increasing frequency and intensity of drought events resulting from global climate change [7–9] is placing further strain on crops and plants in natural ecosystems. Understanding the transcriptional, metabolic, and physiological aspects of drought responses in plants is therefore of critical importance.

Drought often causes reductions in leaf water potential (Ψ) whereby plants initially respond by closing their stomata, and reducing stomatal conductance (*g*_s_) and transpiration (*E*) [2]. While reduced stomatal conductance may limit net photosynthesis (*A*_CO2_) during drought, intense water deficits can also trigger down-regulation of the entire photosynthetic apparatus [10]. These changes limit whole-plant C fixation and growth, and may lead to carbon starvation [11, 12]. Stomatal closure can also limit transpirational cooling and increase leaf temperature, forcing plants to defend against oxidative damage [10, 13, 14]. Stomatal responses to drought stress are often mediated by signaling pathways including Abscisic Acid (ABA) [2, 15, 16]. Despite our understanding of drought response physiology we lack basic information regarding the genetic mechanisms underlying the regulation of plant metabolism and gas-exchange during drought and recovery from drought [15, 17, 18].

Recent studies using microarrays and RNA-sequencing have identified thousands of genes associated with drought stress responses in plants [19–26]. These studies have generally found down-regulation of genes associated with photosynthesis and metabolism, and up-regulation of stress response genes. Regulatory genes including members of the ABA signaling pathway are differentially expressed during drought stress in many species [20, 27–29]. However, little is known about how these gene expression responses are related to physiology and metabolism during drought stress and recovery [20].

*Panicum virgatum* L. (switchgrass) is a C_4_ NAD-malic enzyme (NAD-ME) type perennial bunchgrass native to the tallgrass prairie of North America [30–32]. Switchgrass is considered a promising biofuel crop due to its high productivity, abundant genetic diversity, and large native geographic range [33–35]. Compared to traditional agricultural crops such as corn (*Zea mays*), *P. virgatum* requires little management and uses resources, especially water, more efficiently: a characteristic important for sustainable bioenergy production [36–39]. C_4_ grasses like *P. virgatum* are also key components of native grassland and agricultural ecosystems [40, 41], but our mechanistic understanding of drought responses in C_4_ grasses, more broadly, remains incomplete.

Our study addresses this gap through an integrative analysis of transcriptional, metabolomic, and physiological responses to drought in *P. virgatum.* Here, we asked 1) how gene expression varies under well-watered, drought, and recovery conditions; 2) how gene expression responses to drought vary with diel light:dark cycles; and 3) how changes in gene expression are related to physiological status and metabolite abundance across treatments.

## Methods

### Plant material

Our study focused on AP13, an accession of the lowland *P. virgatum* cultivar Alamo. This cultivar was originally collected in George West, TX in 1972 and released from the James E. “Bud” Smith Plant Material Center near Knox City, TX in 1978 (NRCS). AP13 is the primary clonal genotype of Alamo used for genomic research in *P. virgatum*, with transcriptome and draft whole genome sequence currently available through the DOE Joint Genome Institute (http://www.phytozome.net/panicumvirgatum). Our analysis of AP13 drought responses therefore establishes a foundation for understanding the functional genomic basis of drought responses in the most widely studied accession of *P. virgatum* and more broadly in other C_4_ grasses.

### Soil and plant water balance

**Figure 1:**
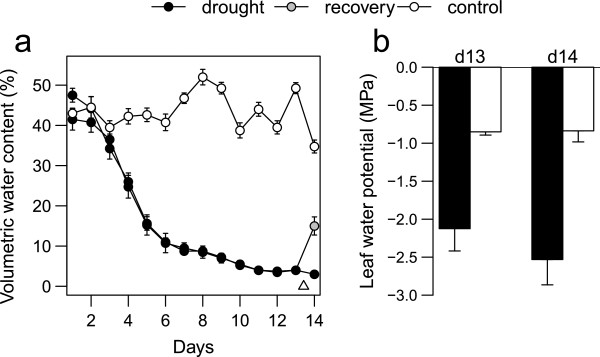
**Effects of drought treatment on (a) volumetric water content of soil and (b) predawn leaf water potential.** △: time of re-watering. Bars and symbols depict mean values, and error bars represent standard error of the mean.

Clonal replicates of *P. virgatum* accession Alamo AP13 (n = 28 plants) were established at the University of Texas at Austin Brackenridge Field Laboratory (BFL) greenhouses in Austin, TX. Plants were propagated by division and independently potted in 3.78 L pots filled with a growth media composed of ProMix (40% sphagnum peat moss, 18% perlite) and a non-swelling clay (Turface, Profile Products, Buffalo Grove, IL), then grown for at least 45 d prior to beginning experiments. For the experiments described here, plants were randomly assigned to either the control group (n = 12), and well watered (1 L day^-1^); or to the drought treatment (n = 16), which received no additional water. Volumetric water content (VWC) of the growth media was measured daily throughout the experiment to monitor soil drying, sampling two locations per pot using a time domain reflectometer (TDR) probe (HydroSense CS620, Campbell Scientific Australia, Garbutt, QLD, Australia). Once VWC fell below 10% in the drought treatment (Figure 1), predawn leaf water potential (Ψ_pd_) was measured using a Scholander-type pressure chamber (PMS Instruments Company, Albany, OR). Previous pot-based studies [42] found that Ψ_pd_ values ≤ -2.0 are associated with ≥50% reductions in net photosynthetic rates in *P. virgatum.* On this basis we chose to begin measurements of gas exchange, gene expression, and metabolism once this threshold (-2.0 MPa) was reached.

VWC in the drought treatment first declined rapidly from 44.9% to 12.6%, then gradually declined to 3.4% by the end of the experiment (day 14). VWC of the well-watered controls remained high throughout the experiment (average = 43.9%). At 10 am on day 14, eight randomly-selected plants from the drought treatment were re-watered with 1 L of water to initiate the “recovery” treatment, increasing VWC in those pots to 16.0% within 4 hours (2 pm). Mature (fully expanded, with clearly defined ligule) leaves were sampled from the upper canopy of each plant at multiple times including pre-dawn and mid-day on days 13 and 14 for measurements of gene expression, metabolite abundance, and physiology. Ψ_pd_ was measured using samples collected pre-dawn (approximately 5:00 AM), while gas-exchange and chlorophyll fluorescence were measured using samples collected mid-day (approximately 2:00 PM). Leaf tissue was preserved for gene expression analysis at each sampling point, and additional samples collected at 10:30 AM and 12:00 PM on day 14 to measure recovery responses. Additional portions of each sampled leaf were stored separately for metabolite analysis. Samples were preserved for gene expression and metabolite profiling by flash-freezing in liquid nitrogen.

### Physiological responses during drought and recovery

Mature upper canopy leaves were sampled from n = 20 plants (6–8 per treatment) for gas-exchange and chlorophyll fluorescence measurements. On day 14, measurements commenced 2 h after initiating the recovery treatment. Leaf net CO_2_ assimilation (*A*_CO2_; μmol m^-2^ s^-1^), stomatal conductance to water vapor (*g*_s_; mmol m^-2^ s^-1^), intrinsic water-use efficiency (*A*_CO2_/*g*_s_ or iWUE; μmol mmol^-1^), photochemical quenching of photosystem II (PSII) (q*P*, dimensionless), and efficiency of PSII (*Φ*_PSII_) were measured on 1–2 leaves using a LI-6400 portable photosynthesis system equipped with a modulated chlorophyll fluorometer (6400–40) integrated into the cuvette lid (LI-COR, Inc., Lincoln, NE, USA). Fluorescence parameters were calculated using built-in functions of the LI-6400 system.

Conditions in the LI-6400 cuvette were set to approximate the ambient growing conditions in the greenhouse. Using an actinic light source, irradiance in the cuvette was set at 1500 μmol m^-2^ s^-1^ photosynthetically active radiation (PAR). Chamber supply [CO_2_] was controlled at 380 μmol mol^-1^, resulting in cuvette [CO_2_] of 373 ± 5.2 (mean ± SD) μmol mol^-1^ across all measurements. The cuvette block temperature was set at ambient and leaf temperature was measured using the LI-6400 leaf thermocouple. Water vapor inside the chamber was not scrubbed such that relative humidity in the chamber approximated ambient conditions. Across sampling points, chamber relative humidity and leaf temperature averaged 64.6 ± 6.1% and 32.5 ± 0.6°C, respectively.

Physiological data were analyzed using a general linear model (ANOVA) with unstructured covariance matrix (to account for the correlations among repeated measurements from the same plants) in SAS PROC MIXED (SAS/STAT v9.2, SAS Institute, Inc.). Effects of measurement day and treatment were tested (alone and in interaction) for leaf water potential data, and effects of treatment for gas-exchange and fluorescence data.

### Transcriptional responses during drought and recovery

Gene expression was profiled at six sampling points throughout the experiment, including both pre-dawn and mid-day sampling times (n = 119 samples; Additional file 1: Table S1). For each sample, RNA was extracted using the Spectrum Plant Total RNA kit (Sigma-Aldrich, Saint Louis, MO, USA) and treated with DNAse I (Sigma-Aldrich) to remove genomic DNA. One μg of intact total RNA per sample was used to prepare cDNA tag libraries as previously described and applied to *Panicum* [43, 44]. Samples were assigned sample-specific oligonucleotide barcodes and pooled for multiplexed sequencing on the SOLiD platform (version 3.0, Applied Biosystems) at the University of Texas, Austin.

cDNA tag libraries prepared from each sample were sequenced at 5.7 million raw reads per sample on the SOLiD platform, 69% of which (high-quality reads, HQ) passed quality and adaptor filters. Prior to analysis, reads were trimmed to remove four non-template bases introduced at the 5′ end of each tag during library preparation and exclude uninformative reads (homopolymer regions ≥10 bases in length, >10 bases with quality scores < 20, or matching adaptors from library construction [cross_match alignment score ≥ 10]).

We first analyzed these data by aligning HQ reads against a recently published *P. virgatum* transcriptome assembly [45], but found that a large proportion of reads matched multiple transcripts in that assembly equally well and therefore had to be excluded. To minimize this data loss, which may have resulted from the inclusion of multiple genotypes in the published assembly, we instead developed a custom transcriptome assembly using exclusively Alamo AP13 data from the same study. Summary statistics of this custom assembly are shown in (Additional file 1: Table S2). Assembled transcripts (isotigs) were annotated with gene names based on BLASTX comparisons with the UniProt database (version 2010_09; e-value ≤ 10^-4^), and with Gene Ontology (GO) terms based on GO annotation of UniProt records (http://www.geneontology.org). To facilitate functional analysis in MapMan [46], transcripts were assigned to functional categories (bins) using Mercator [47] based on sequence similarity with annotated reference sequences (TAIR release 9, UniProt plant proteins, KOG, CDD, and TIGR rice proteins).

The Roche De Novo Assembler used for our custom assembly tracks relationships among contigs to organize isotigs (transcript models) into isogroups intended to represent the collections of transcripts from a single locus. In the tetraploid genome of Alamo AP13, these isogroups are expected to combine homeologs which generally show little sequence divergence (<2%) [30]. However, RNA-Seq data would be ineffective at discriminating between homoelogs for the same reason, regardless of reference, and since any functional differences between homeologs remain unknown, the functional interpretation of our expression data would be unaffected in any case. We therefore chose to filter for ambiguity and count matches for expression analysis at the isogroup level.

HQ reads were aligned in color-space against the custom AP13 assembly using SHRiMP alignment software (version 2.1.1b) [48], running *gmapper-cs* with options ‘--strata -o 10 -N 16′. Alignments were filtered with *probcalc* to eliminate weak matches (*P*_chance_ > 0.05), and short (<35 bp aligned, or < 32 matching bp) or ambiguous alignments removed with custom Perl scripts. 59% of HQ reads were unambiguously mapped to a single isogroup, yielding on average 2.6 million mapped reads per sample for statistical analysis. Rarefaction analysis (Additional file 1: Figure S1) showed that this sequencing depth captured the majority of transcripts (85%) detected at >10-fold higher sequencing depths (28 million mapped reads).

Statistical comparisons of RNA-Seq count data typically use negative binomial models well suited for the over-dispersed counts data characteristic of RNA-Seq [49]. However, currently available software implementing this approach does not model random factors as required for ‘repeated measures’ analysis. To balance these concerns, we transformed counts data using a variance stabilizing procedure *voom* in the R module *limma* [50] designed to transform count data from RNA-Seq into weighted expression values suitable for linear modeling. Individual (plant) was modeled as a random factor to account for correlation among repeated measurements. Differential expression was tested using an empirical Bayes method function (*eBayes*), with false discovery rate (FDR) controlled at 0.05.

To evaluate transcriptional responses to drought in the context of diel light:dark cycles, we compared stressed and control treatments (n = 74) sampled pre-dawn and mid-day on days 13 and 14. To investigate transcriptional responses during recovery from drought stress, a nested set of samples (n = 58) were collected from the same plants on day 14 (0.5, 2, and 4 hours after re-watering) for all three treatments (drought, control, and recovery).

### Functional analysis of responses to drought and re-watering

To identify metabolic pathways and processes responding to drought stress or recovery, expression changes in each functional category (MapMan bin) were compared to the overall responses across all genes (Wilcoxon rank sum tests, FDR = 0.05). Effects of drought were evaluated by comparing the average difference between drought and control treatments across all sampling points. The effects of recovery were evaluated by comparing the average difference between recovery and drought treatments across all sampling points following re-watering.

To evaluate expression changes relevant for C_4_ photosynthesis we selected genes associated with this process based on Mercator annotations of our transcriptome data and previously published descriptions of C_4_ photosynthesis in grasses [51]. To integrate expression and metabolite data for this pathway, fold-changes in gene expression and metabolite abundance were calculated based on the subset of plants that were sampled for both analyses.

### Validation of expression profiles by qPCR

Comparisons between qPCR and RNA-Seq were performed using four replicates from each treatment at pre-dawn (drought and control) and mid-day (drought, control, and recovery) sampling points on day 14 (n = 20 samples). Oligo-dT primed (dT_20_) first-strand cDNA was prepared for each sample using 500 ng total RNA and Superscript II reverse transcriptase (Clontech, Mountain View, CA, USA), then used for duplicate qPCR reactions for each sample and target. RT-qPCR was conducted with SYBR Green PCR Master Mix (Invitrogen, Carlsbad, CA, USA) using a 7300 Real-Time PCR System (Applied Biosystems). Primer efficiency was verified using a cDNA dilution series (100% ± 5%) and specificity by melt curve analysis. Stable expression of reference genes was verified based on replicate samples (n = 4 from each group) with equal amounts of total RNA in each reaction analyzed using the 2^-ΔCt^ method, and expression values normalized to the average C_t_ of three stable reference genes (*CoxI*, *CyCTI-3*, and *Eif5a*) using the ddC_T_ method [52].

### Metabolomic consequences of drought stress

To complement the expression profiling data, additional samples were collected from a subset of plants (four from each of control, stressed, and recovering) at the end of the experiment and shipped on dry ice to the Metabolomics Central Service Core Laboratory at University of California, Davis. Gas chromatography and time-of-flight mass spectrometry were used to quantify small molecules involved in primary metabolism, and individual compounds identified from mass spectra and annotated using BinBase [53]. Raw metabolomic data are provided in supporting information (Additional file 2: Table S3). For statistical comparisons between treatments, metabolite abundance data were log-transformed and scaled to the average value in control samples. Transformed abundance data were compared using ANOVA, with FDR controlled at 0.1.

### Relationships between gene expression, metabolomics, and physiology

Linear correlations between gene expression and metabolite abundance were based on weighted expression data (RNA-Seq) and the log-transformed abundance of each metabolite in the same samples (n = 12). The larger sample size available for physiological traits (n = 32) made it possible to search for both linear and non-linear relationships between gene expression and physiology using maximal information coefficient (MIC) as implemented in the MINE software [54]. Significance of these relationships was evaluated using pre-computed *P*-values from MINE, with Bonferroni correction for multiple tests.

### Availability of supporting data

The custom transcriptome assembly used as a reference in this study is available at the Dryad data repository (doi:10.5061/dryad.6630k). RNA-Seq data are available at NCBI’s Gene Expression Omnibus (Series GSE57887).

## Results

### Physiological effects of drought and recovery

The reduced soil water content imposed by the drought treatment (Figure 1a) caused visible indications of stress by day 13, at which point ~50% of plants showed leaf yellowing and rolling, but not senescence. Pre-dawn leaf water potential (Ψ_pd_) declined accordingly (Figure 1b), falling below -2.0 MPa in the drought treatment on day 13 (mean ± SE = -2.1 ± 0.3 MPa) while remaining significantly higher in controls (-0.85 ± 0.04 MPa; *P* = 0.001). Similar effects were found on day 14 (drought Ψ_pd_ = -2.5 ± 0.3 MPa; control Ψ_pd_ = -0.84 ± 0.15 MPa); no effects of sampling day (13 vs. 14) or day × treatment interactions were observed (*P* = 0.53 and 0.51, respectively).

**Figure 2:**
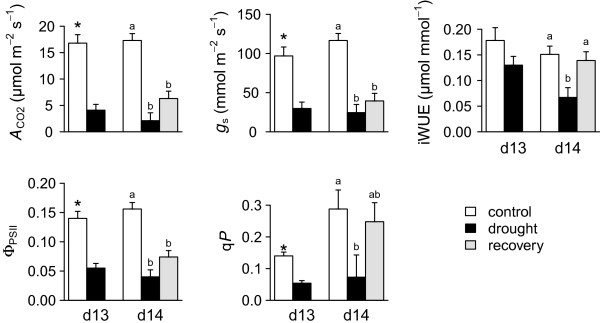
**Effects of drought and recovery treatments on photosynthesis and gas exchange.** Values shown are mean ± standard error. *A*_CO2_: light saturated net photosynthetic rate; *g*_s_: stomatal conductance; iWUE: intrinsic water-use efficiency; *Φ*_PSII_: efficiency of photochemical quenching; q*P*: photochemical quenching. *indicates significant differences between drought and control treatments on d13 (at which point these were the only treatment). Significant differences at d14 (comparing three treatments) are indicated by lowercase letters; bars sharing a letter were not significantly different.

Gas exchange rates and photochemical traits also declined substantially in the drought treatment relative to controls (*P* < 0.05; Figure 2). *A*_CO2_ and q*P* declined 5.5 and 3.4 fold, respectively, in drought plants relative to controls. Similarly, stomatal conductance (*g*_s_) was reduced 3.9-fold in the drought treatment relative to controls (Figure 2). Because the reductions in *A*_CO2_ outpaced reductions in *g*_s_, iWUE was slightly lower in the drought treatment. Although this trend was not significant on day 13 (*P* = 0.14), a significant difference was detected on day 14 (*P* = 0.01; Figure 2).

Although several gas exchange and fluorescence traits showed a slight increase after rewatering (Figure 2), these trends were not significant for most traits. Interestingly, although *g*_s_ and *A*_CO2_ did not return to control levels after rewatering, their ratio (iWUE, water use-efficiency) returned to nearly control levels (0.14 and 0.16 μmol mmol^-1^ for recovering and control, respectively). This occurred rapidly (<4 hr), even though water availability (VWC) had not yet returned to control levels (Figure 1a). These rapid physiological responses demonstrate the plasticity of gas exchange in switchgrass, highlighting a potentially adaptive trait in water limited habitats.

### Expression profiling drought and recovery using RNA-seq

**Figure 3:**
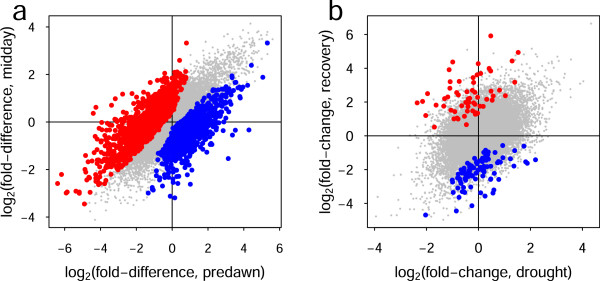
**Interactions between drought/recovery treatments and circadian patterns.** Each symbol depicts a single gene, with significant treatment × time interactions highlighted in red (up-regulated in contrast shown on y-axis) or blue (down-regulated). **(a)** Effects of the drought treatment (fold change in drought relative to control) in predawn and midday samples. **(b)** Expression changes during recovery, and changes during the same period in drought treatment.

Gene expression profiling of drought responses revealed that a large fraction of the transcriptome (37.2%) was differentially expressed in the drought treatment relative to controls (Table 1). While a comparable fraction of the transcriptome was affected by the treatment at both pre-dawn and mid-day timepoints, different genes were affected by the treatment depending on the time of day. Overall, the effects of drought varied as a function of time of day for 2,365 transcripts. These time × treatment interaction effects can be visualized by comparing the fold-change across time points (Figure 3a). While many of the genes affected by drought treatments showed similar responses at both sampling times, 1,229 were up-regulated in mid-day samples but down-regulated or stable in pre-dawn samples (e.g. isogroup03982, a homolog of starch synthase). Many genes (1,136) showed the opposite pattern; e.g. isogroup32485 (a homolog of wound-induced protein) was up-regulated in drought during pre-dawn and down-regulated in drought during mid-day (Figure 3a).

**Table 1:** **Differential gene expression in drought and recovery treatments as a function of treatment, time of day, and their interaction**

Comparison	Source	Contrast	DEG (n)
Drought (n = 74)	Treatment	Drought – Control	10,180
	Time of day	2:00 PM – 5:00 AM	9,045
	Treatment × time	d_2PM_ – d_5AM_	2,365
Recovery (n = 58)	Treatment	Recovery – Drought	1,514
	Time of day	2:00 PM – 10:30 AM	2,196
	Treatment × time	d_REC_ – d_STR_	148

Normalized expression data compared using a linear model with individual as a random effect, and treatment and time as fixed factors.

d_5AM_, d_5AM_: treatment effects at 5:00 AM and 2:00 PM, respectively.

d_REC_, d_STR_: change in gene expression between 10:30 AM and 2:00 PM for recovering and stressed plants, respectively.

**Figure 4:**
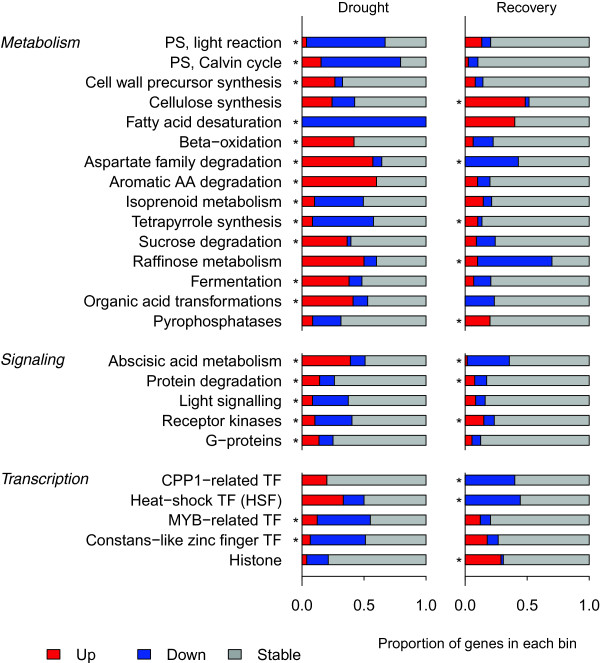
**Functional categories responding differently in drought stress and recovery treatments.** In each MapMan pathway shown (*metabolism overview*, *regulation overview*, and *transcription*), bins that differed from the overall response in either treatment are listed. Within each bin, the proportions of genes up- or down-regulated by ≥50% are coded red and blue respectively. *: significant differences, Wilcoxon rank-sum test, FDR = 0.05.

We identified rapid transcriptional responses after re-watering in the recovery treatment. Many transcripts (1,514) were differentially expressed at one or more sampling points during recovery relative to the drought treatment (Table 1). A slightly larger fraction of the transcriptome (2,196 transcripts) was differentially expressed between sampling points independent of treatment. More than one hundred genes showed significant interaction effects, 60 of which were up-regulated in recovering plants but stable or down-regulated in drought (e.g. isogroup24130, a putative citrate transporter). The remaining 88 transcripts showed the opposite pattern; e.g. isogroup10027, a homolog of the tonoplast dicarboxylate transporter, was down-regulated in recovering plants but up-regulated in the drought treatment during the same period (Figure 3b). Because the same plants were sampled repeatedly for these measurements, we cannot exclude the possibility that differences in gene expression may reflect specific treatment × sampling effects (i.e., the effects of wounding on gene expression could in principle depend on plants’ physiological condition). A complete list of differentially expressed genes (DEG) along with their statistics and annotation is provided in Additional file 3: Table S5, and the raw expression data (number of reads mapping to each isogroup) in Additional file 4: Table S6.We identified multiple metabolic processes affected during drought and recovery through functional analysis of expression profiles with MapMan (Figure 4). Many photosynthetic genes were down-regulated in the drought treatment, including light reaction and Calvin cycle genes. Other processes that were up-regulated in the drought treatment include sucrose degradation, fermentation, and organic acid transformations. These drought-associated processes responded only slightly during the recovery. However, other processes responded rapidly during recovery, reversing the gene expression changes originally induced by drought. For example, aspartate family amino acid degradation genes were up-regulated during drought and rapidly down-regulated during recovery.Regulatory and cell signaling pathways also showed contrasting responses in drought and recovery (Figure 4). Genes associated with ABA metabolism were up-regulated in drought plants but down-regulated during recovery. Several families of transcription factors (TFs) were affected by drought or recovery treatments (Figure 4), including some genes with sharply contrasting responses to these treatments. For example, transcripts homologous to CPP1 (a transcription factor associated with root nodule development) and heat shock TFs were down-regulated during recovery, but not during drought. MYB-related and Constans-like zinc finger TFs, in contrast, were down-regulated in the drought treatment, but not during recovery.

### Validation of expression profiles by qPCR

**Figure 5:**
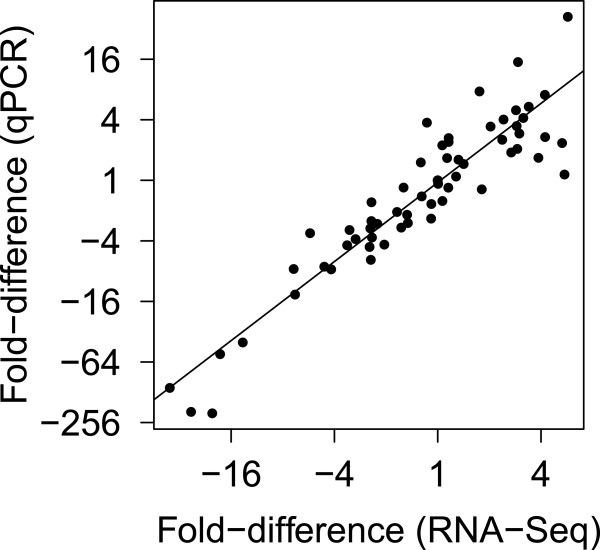
**Validation of RNA-Seq expression profiles using qPCR.** Each symbol depicts fold difference in gene expression relative to mid-day control samples for a single gene and sample, relative to internal reference genes. Three technical replicates were conducted for each qPCR of 15 genes in n = 20 samples and compared with normalized RNA-Seq data from the same samples.

qPCR was used to validate expression changes observed in RNA-Seq data for a panel of 15 differentially expressed genes (DEG; Additional file 1: Table S4), using stable genes identified in RNA-Seq as internal reference genes (*CoxI*, *CyCTI-3*, and *Eif5a*) (Additional file 1: Figure S2). A subset of RNA samples (n = 20) from day 14 were selected for validation, including pre-dawn and mid-day samples from both drought and control treatments as well as mid-day samples from the recovery treatment. This analysis showed close agreement between fold-changes in gene expression as measured by qPCR and RNA-Seq (*r* = 0.93; Figure 5). Detailed comparisons by gene and treatment are shown in Additional file 1: Figure S3.

### Metabolomic consequences of drought stress

**Figure 6:**
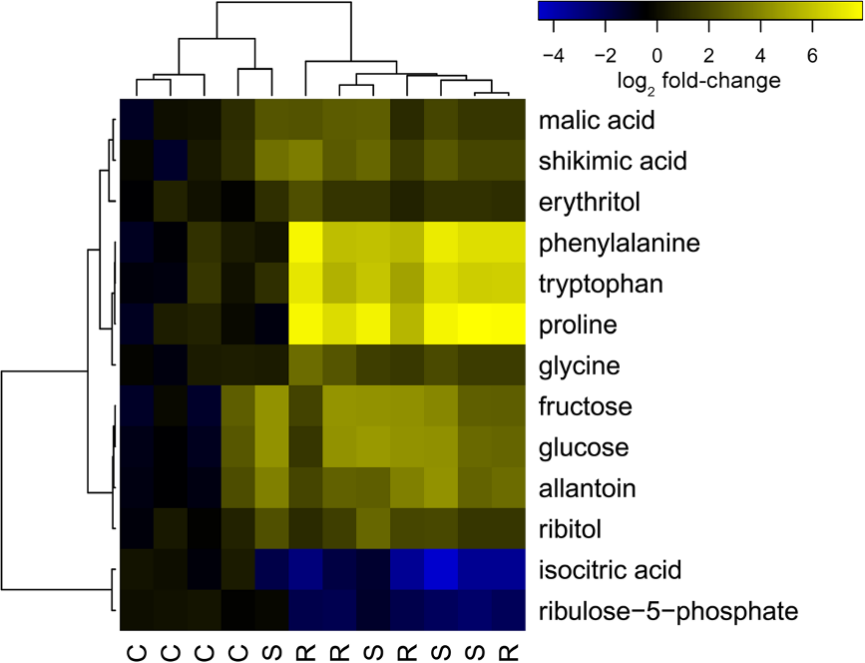
**Differences in primary metabolite profiles during drought and recovery.** Metabolites significantly affected by treatment are shown. Heatmap colors indicate log-transformed abundance of each metabolite relative to controls. C: control; S: drought-stressed; R: recovery.

We profiled metabolites in a subset of samples (mid-day on day 14) to characterize metabolic consequences of drought and provide a functional context for our gene expression analysis. Approximately one third (n = 144) of the 405 peaks in mass spectra (MS) were identified based on comparisons to known compounds. These identified peaks accounted for a majority (76.7%) of the total MS signal. Because the total size of metabolite pools differed significantly between treatments (*P* = 0.012), MS data for each compound were scaled to the average signal in controls, rather than the total from each sample. Because of the relatively small number of samples and large number of tests conducted, we chose a relaxed FDR threshold of 0.1 (i.e. approximately 10% of differences are expected to be type I errors). Analysis of the log-transformed, scaled data revealed that the abundance of 13 primary metabolites was significantly affected by the drought treatment at this relaxed threshold. Most of these compounds were enriched during the drought relative to the control, including amino acids (>32-fold), monosaccharides (>14-fold), and organic acids (>4-fold) (Figure 6). Ribulose-5-phosphate and isocitric acid, in contrast, were depleted (3- and 7-fold, respectively) during drought relative to controls. No differences between recovery and drought treatments were observed, perhaps simply as a result of the short duration (~4 h) of the recovery treatment.

### Integrative analysis of gene expression, metabolomics, and physiology

We identified 328 genes significantly associated with physiological traits using MIC analysis (Bonferroni-adjusted *P* < 0.05). These relationships are summarized in Table 2, and a complete list is provided in Additional file 5: Table S7. We detected approximately equal numbers of positive and negative relationships (52 and 48% respectively). Most relationships were non-linear (|*r*| < 0.80 for 72% of significant associations), highlighting the value of MIC analysis for delineating relationships between gene expression and physiological traits.

**Table 2:** **Relationships between gene expression and physiology identified using maximal information coefficient (MIC)**

Physiological trait	Linear		Nonlinear	
	Positive	Negative	Positive	Negative
Ψ_pd_	39	57	88	74
*A*_CO2_	0	0	1	3
*g*_s_	2	0	9	5
*Φ*_PSII_	3	0	11	5
q*P*	0	1	25	26

Numbers of genes with significant relationships (Bonferroni-adjusted *P* < 0.05) are shown for each trait. Relationships with Pearson’s correlation coefficient *r* ≥ (±) 0.8 classified as linear.

While linear correlations can be simply classified as positive or negative, non-linear relationships may include diverse types of functions. Two different patterns (relationships between gene expression and physiology) were apparent in our findings.

**Figure 7:**
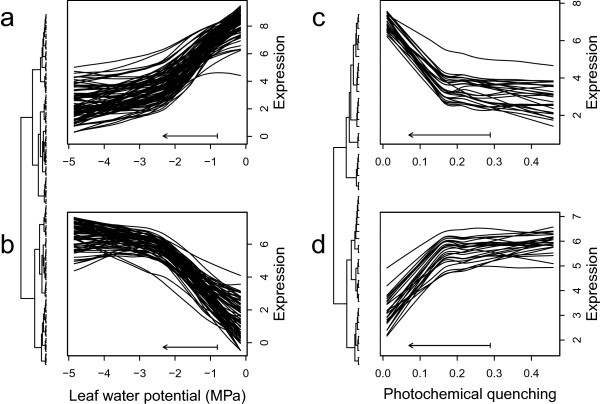
**Non-linear relationships between gene expression and physiological traits.** For each trait, all transcripts with significant non-linear relationships are shown (MIC *P* < 0.05 after Bonferroni correction, |*r*| < 0.8). Transcripts showing similar patterns were grouped by hierarchical clustering of dissimilarity matrices. Each line represents a series of paired expression data and physiological measurements smoothed using local polynomial regression (span = 0.8). Arrows indicate the direction of change during drought, from the average in control plants (base of arrow) to the average in stressed plants (arrowheads). **(a, b)** n = 162 transcripts showing non-linear relationships with predawn leaf water potential. **(c, d)**  = 51 transcripts showing non-linear relationships with photochemical quenching. Panels **a** and **d** depict genes expressed at higher levels in the control than the drought treatment, and vice versa for panels **b** and **c**.

In the first relationship (Figure 7a, b), expression and physiology were initially tightly coupled as Ψ_pd_ declined from control levels (base of arrow in Figure 7a; -0.85 MPa). Expression of some genes decreased as Ψ_pd_ decreased (Figure 7a; n = 128), while expression of others increased (Figure 7b; n = 124). Once a threshold level of Ψ_pd_ was reached (approximately -2.5 MPa), expression became decoupled from Ψ_pd_ and remained constant despite continued declines in Ψ_pd_. The set of genes responding to Ψ_pd_ in this fashion was enriched for inorganic cation transport (GO:0015672) and dicarboxylic acid metabolism (GO:0043648) (Fisher’s exact test; adjusted *P* = 0.024). Complex patterns of regulation were observed within both functional categories, with some genes upregulated during drought stress (e.g. isogroup06639, a putative malic enzyme homolog, and isogroup03586, a putative sodium/hydrogen exchanger) and others down-regulated (e.g. isogroup11673 [ATP synthase, gamma chain] and isogroup19577 [malate dehydrogenase]). Notably, several of the genes in this latter category have known roles in C_4_ photosynthesis.

In the second pattern we observed, expression was initially decoupled from physiology (in benign control conditions), but responded strongly to changes in physiology below a threshold value. This pattern is best exemplified by q*P* (Figure 7c, d). Gene expression remained constant as q*P* declined from control values of ~0.29, until a threshold value was reached (~0.17). Beyond this threshold, gene expression declined sharply, with further reductions in q*P* for 27 genes (Figure 7c). Another 27 genes increased with declining q*P* after the same threshold was reached (Figure 7d). The set of genes responding to qP in this fashion was significantly enriched for monosaccharide metabolism (GO:0005996) (adjusted P = 0.028), expression of which decreased during drought stress. Similar responses were observed for *g*_s_, suggesting a threshold of approximately 70 mmol m^-2^ s^-1^ (Additional file 1: Figure S4). The observation that many genes show abrupt changes in expression across the same narrow range of physiological conditions suggests that these may represent fundamental thresholds in drought stress response.

We identified strong linear relationships between gene expression and metabolite abundance (Pearson’s correlation coefficient |*r*| > 0.9), including 83 genes associated with metabolites affected by the drought treatment (Table 3). Many of the relationships identified in this analysis would not have been predicted based on sequence similarity alone. For example, expression levels of 28 genes were correlated with shikimic acid, approximately equally distributed among positive and negative correlations. The list of correlated genes includes metabolic enzymes that, although not directly implicated in shikimate synthesis, may be related to changes in abundance of precursors or products of these pathways (e.g., dehydrogenases, glycosyltransferases; Additional file 6: Table S8). Sequence homology suggests regulatory roles for other genes correlated with shikimate abundance (e.g. protein phosphatases and kinases; Additional file 6: Table S8). Overall, 110 of the 144 identifiable metabolites were associated with one or more genes. A small fraction of the transcriptome was implicated by this analysis (n = 341 genes), and most of these associations were highly specific (89% of genes were each associated with a single metabolite).

**Table 3:** **Relationships between gene expression and metabolite profiles**

	Correlated genes (n)	
Metabolite	Positive	Negative
Allantoin	3	1
Erythritol	1	1
Fructose	5	4
Glucose	3	3
Glycine	1	1
Isocitric acid	0	1
Malic acid	10	5
Phenylalanine	1	0
Proline	1	0
Ribitol	7	4
Ribulose-5-phosphate	0	1
Shikimic acid	13	15
Tryptophan	2	0

Statistics shown only for those metabolites showing significant treatment effects (ANOVA; *P* < 0.05) and significant linear relationships with gene expression (|*r*| ≥ 0.9).

In total, we identified 661 genes associated with physiological traits or metabolite abundance. A set of 23 putative transcription factors associated with physiology or metabolites in this analysis present especially promising candidates for future studies of transcriptional regulation during drought and recovery (Additional file 1: Table S9).

**Figure 8:**
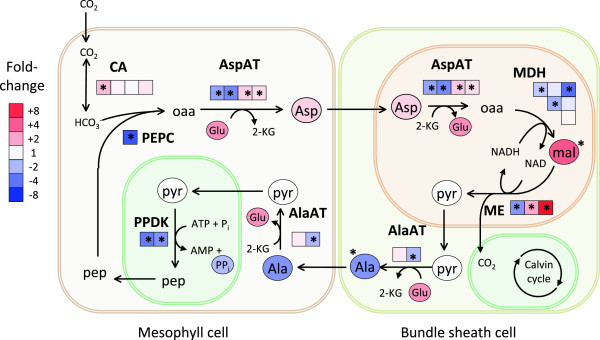
**Changes in C**_**4**_ **photosynthetic processes during drought stress.** NAD-ME pathway redrawn from (Maier *et al.*, [51]). Colors of metabolites (ovals) and transcripts (squares) indicate fold differences in stressed plants relative to controls. Multiple transcripts matching each gene are ordered by expression (highest to lowest from left to right); transcripts expressed at low levels (<20% of most highly expressed transcript for each gene) are omitted for visual clarity. *: transcripts or metabolites significantly affected by treatment. AlaAT: alanine aminotransferase; AspAT: alanine aminotransferase; CA: carbonic anhydrase; Mal: malate; MDH: malate dehydrogenase; ME: malic enzyme; PEPC: phosphoenolpyruvate carboxylase; PPDK: pyruvate orthophosphate dikinase; Pyr: pyruvate.

The combined analysis of gene expression and metabolite abundance allowed us to examine in detail how components of photosynthesis were impacted by drought. Many genes associated with C_4_ photosynthesis were down-regulated in the drought treatment (Figure 8), including alanine and aspartate aminotransferases (AlaAT, AspAT), malate dehydrogenases (MDH), one of the NAD-malic enzyme homolog (ME), pyruvate orthophosphate dikinases (PPDK), and phosphoenolpyruvate carboxykinase (PEPC). In contrast, one carbonic anhydrase gene was significantly up-regulated during drought and other CA genes trended upward. Multiple transcripts homologous to each gene in these pathways were observed, and in some cases these showed contrasting responses. Most notably, the ME homolog isogroup00615 was down-regulated 2.8-fold, while isogroup06639 was up-regulated 11.7-fold. Since compartmentalization of cellular functions is an important aspect of C_4_ adaptations [55], these contrasting responses probably reflect cell- or tissue-specific expression patterns. Further studies will be needed to identify the cells and tissues in which these responses occur during drought stress. Several C_4_ metabolic intermediates showed a trend toward depletion in drought (pyruvate, alanine, and pyrophosphate), although these differences were not significant. Malic acid, in contrast, was significantly enriched in the drought treatment (4.2-fold) relative to controls. No significant changes in expression or metabolite abundance for these pathways were apparent in the recovery treatment, except for a single CA transcript (isogroup00318) down-regulated 1.6-fold. All details of gene expression and metabolite changes for C_4_ pathways are shown in Additional file 7: Table S10.

## Discussion

Our study examined drought responses across multiple levels of biological organization in a perennial C_4_ grass, *P. virgatum* (switchgrass)*.* Drought treatments produced extensive changes in gas exchange and photosynthetic physiology, metabolite profiles, and gene expression. We identified non-linear relationships between gene expression and leaf physiology that suggest discrete thresholds at which gene expression changes abruptly during drought stress. We also identified corresponding changes in gene expression and metabolite profiles associated with the C_4_ carbon fixation cycle. These findings provide new insights into the mechanisms of drought stress response in *P. virgatum* and establish a baseline for studies of natural variation in drought responses among diverse accessions.

### Drought responses and recovery

Gas-exchange and chlorophyll fluorescence were strongly reduced in the drought treatment as expected. Previous studies in *P. virgatum* have found similar responses [42, 56] with gas-exchange and photosynthetic traits declining during drought. As in other C_4_ species, leaf yellowing observed in the drought treatment may reflect N retranslocation out of the leaves [57], which may constrain physiological recovery from drought. Consistent with previous studies of gene expression responses to drought, [19, 25, 29, 58, 59], we found that genes involved with photosynthetic light reactions (PSI) and carbon fixation (PSII) were down regulated in the drought treatment. This may reflect down-regulation of the photosynthetic apparatus to match substrate (e.g. ATP) availability [2, 60, 61]. However, drought stress can also result in expression of sugar-responsive genes that suggest increased, rather than decreased, substrate availability [62]. Consistent with this possibility, many genes associated with sugar degradation and fermentation were up-regulated (Figure 4) and monosaccharides accumulated (Figure 6) during drought. This suggests that plants may catabolize cellular C reserves to avoid short-term C limitations and so preserve cellular function during drought. Alternatively, the up-regulation of sugar metabolism genes and accumulation of monosaccharides may reflect leaf osmotic adjustment, since many sugars act as osmolytes in drought stress responses [15, 17].

Our findings are consistent with *P. virgatum* responses to drought being influenced by the ABA signal transduction pathway, as documented in other plants [2, 15–17]. While this signaling pathway can clearly trigger a wide range of physiological responses including stomatal closure, stomatal closure may also result from physical changes in the transpiration stream, and ABA could simply be a regulator of drought recovery and adaptation [63].

The observed changes in gene expression and metabolism also highlight the multiple stresses imposed by drought. For instance, the stomatal closure brought on by drought not only limits C fixation but also transpirational cooling, potentially leading to thermal stress and oxidative damage. Drought-induced down-regulation of PSII affects electron partitioning, redirecting electrons from use in photosynthesis to the dissipation of excess light energy and production of harmful reactive oxygen species (ROS). ROS can oxidize amino acids and proteins resulting in damage to cells and the photosynthetic apparatus as a whole [10, 17]. Correspondingly, we observed that several beta-oxidation and heat shock protein genes were up-regulated in the drought treament, which suggests potential responses to thermal stress and oxidative damage [40, 64, 65]. Other drought studies have found similar expression of genes related to thermal defense [58, 59] and reactive oxygen species (ROS) detoxification [66].

The controlled conditions under which our experiment was conducted suggest caution in generalizing these findings to field conditions. The rate of soil drying in small (3.78 L) pots may be faster than in native soil or agricultural settings in which soil water availability can be strongly affected by neighboring plants. Likewise, the short-term recovery treatment in our experiment may not be representative of the long-term impacts of drought in field conditions.

### Gene expression responses to drought and the diel cycle

Regardless of other environmental influences such as water availability, gene expression profiles are profoundly affected by the diel light:dark cycle [67]. Recent studies have begun to consider how diel effects may interact with drought stress responses [68, 69], finding that transcriptional responses to drought treatments depend strongly upon time of day. In *Arabidopsis*, an order of magnitude more genes were affected by time of day (7,429) than by drought treatments (759) [68]. This contrasts with our findings in which a comparable number of genes were affected by time of day (9,045) as by the drought treatment (10,180). Further, many more genes were affected by treatment × time interactions in our study (2,365) than in previous studies of *Arabidopsis* (4) [68]. These contrasting findings may reflect differences in experimental drought treatments, expression profiling platforms, or taxon-specific responses. Consistent with these studies, our findings suggest that evaluating drought responses at a single time of day would grossly underestimate the scope of transcriptional responses to drought. Future studies of drought response in C4 grasses and other plants may benefit from sampling at multiple time points, and at minimum, the precise time of sampling should be reported to facilitate comparisons across studies. Interestingly, similar work in Poplar suggest these interactions between diel and drought effects depend on genotype [69]. In that study, transcriptional responses to drought peaked at different times of day in two different commercially important clones. While the present study focused on a single switchgrass genotype, this observation suggests that interactions between diel and drought effects may be similarly important in shaping responses of switchgrass to drought stress and should be considered in future studies of diverse switchgrass accessions.

### Integrating transcriptional, metabolomic, and physiological responses

Although our experimental design consisted of only two treatments (watered and unwatered), variation in application of these treatments or the rate of soil drying among pots, or water use efficiency among plants, produced a continuous distribution of variation in drought stress.

For example, Ψ_pd_ ranged from -4.8 to -0.6 in the drought treatment and from -1.5 to -0.2 in controls (Figure 1b). This variation provided an opportunity to search for correlations between gene expression and other phenotypes. We uncovered non-linear relationships between gene expression and physiological traits, suggesting thresholds in leaf physiological status that may drive important transcriptional changes during drought stress. This pattern was especially clear for Ψ_pd_ (including genes involved with inorganic cation transport, and metabolism of malate and other dicarboxylic acids) and q*P* (including genes associated with monosaccharide metabolism). Accumulation of inorganic cations during drought may reflect osmotic adjustments [70–72], but inorganic cations may also serve to balance organic acids such as malate [71]. Malate has often been associated with stress responses in plants and is usually associated with changes in stomatal conductance, osmotic potential, or photosynthetic capacity [73, 74]. Malate plays an important functional role in photosynthesis for many C_4_ plants where it is decarboxylated, leading to a release of CO_2_ into the bundle sheath, which is then used in the Calvin cycle [55]. The relationships between gene expression and physiology (Figure 7) suggest that regulation of C_4_ gene expression and the abundance of metabolic intermediates (Figure 8) are highly sensitive to small deviations from typical Ψ_pd_ values, but that once the threshold (~ -2.5 MPa) is reached, further decreases have no effect on gene expression. Future studies of variation in drought tolerance among *P. virgatum* accessions and under stress imposed under more natural field conditions will be important for exploring variation in these thresholds.

Interpretation of the relationship between monosaccharide genes and q*P* (the proportion of open PSII reaction centers in the light-harvesting antennae of the thylakoid membrane) is less clear. q*P* generally provides information on processes affecting photochemical efficiency [75]. During drought stress, soluble sugars often accumulate [76] and serve multiple functions include signaling and osmotic adjustment [60, 77–79]. Our observation that monosaccharide metabolism genes (including several glycolytic enzymes) are down-regulated during drought stress is consistent with these roles and with the observed accumulation of monosaccharides (glucose, fructose) in the drought treatment.

Clearly, the relationships (both linear and non-linear) between gene expression and other phenotypes identified in this study are only correlations. Further research will be needed to clarify the causal relationships among these variables.

### Variation in drought responses in a changing climate

Climate models predict an increasing frequency and intensity of drought events during the next century [7, 9]. Considering the central role of drought stress in structuring plant communities, these projections highlight the importance of understanding variation in drought stress responses, including drought recovery, within and among plant taxa. *Panicum virgatum* occurs naturally across a wide precipitation gradient [80–84], and while some studies have found little physiological variation among populations in response to drought [42, 56], other studies including diverse genotypes have shown extensive variation in physiological responses to variable soil moisture (Aspinwall *et al.*, in review). Exploring variation in physiological and transcriptional responses to soil moisture availability among *P. virgatum* cultivars and populations will provide additional insight into the mechanistic basis of these differences. Examining whether genotypes differ in the timing (physiological thresholds at which expression changes are induced) or magnitude of gene expression responses during drought stress may be especially informative.

## Conclusions

Overall, our results provide a new perspective on the complex mechanisms underlying drought stress responses in plants. Further studies describing the mechanistic basis for natural variation in drought tolerance will be important for understanding the scope of plant drought tolerance and adaptation, and may advance the development of drought-tolerant germplasm required for agricultural sustainability under climate change.

### Ethics

All research was carried out in accordance with institutional, local, and federal regulations. For this greenhouse-based study of a widely cultivated crop species, no special ethical consent or approval was required.

## Electronic supplementary material

Additional file 1: **All supplementary figures and tables except for large data tables, which are provided separately (below).** This document includes **Figures S1-S4, and Tables S1, S2, S4, and S9**. (DOC 2 MB)

Additional file 2: Table S3: Raw metabolite abundance data. (XLS 44 KB)

Additional file 3: Table S5: Complete list of DEG for all contrasts in both drought and recovery experiments. (XLS 4 MB)

Additional file 4: Table S6: Raw gene expression data (number of reads mapped to each isogroup). (ZIP 5 MB)

Additional file 5: Table S7: Complete list of relationships between physiology and gene expression identified using maximal information coefficient. (XLS 82 KB)

Additional file 6: Table S8: Complete list of genes showing linear correlations between gene expression and metabolite abundance. (XLS 76 KB)

Additional file 7: Table S10: Expression changes in genes associated with C_4_ photosynthesis. (XLS 3 MB)
