# Supplementary material for: Integrating transcriptional, metabolomic, and physiological responses to drought stress and recovery in switchgrass (Panicum virgatum L.)
Source: BMC Genomics. 2014 Jun 26;15(1):527. doi: 10.1186/1471-2164-15-527 (PMC4122788; doi:10.1186/1471-2164-15-527)
Supplement: Supplementary file 1 — Additional file 1: All supplementary figures and tables except for large data tables, which are provided separately (below). This document includes Figures S1-S4, and Tables S1, S2, S4, and S9. (DOC 2 MB) [file 12864_2014_6310_MOESM1_ESM.doc]

**Additional file 1**

Integrating physiological, transcriptional, and metabolomic responses to drought stress and recovery in switchgrass (*Panicum virgatum* L.)

Table of contents

Table S1. Tissue sampling and sequencing depth for RNA-Seq analysis of drought and recovery treatments

Table S2. Details of custom transcriptome assembly used as a reference for RNA-Seq analysis.

Figure S1. Rarefaction analysis of gene detection with increasing sequencing depth.

Table S4. Targets, primer sequences, and efficiency data for qPCR primers used in this study.

Figure S2. Experimental validation of stability for qPCR reference genes.

Figure S3. Details of qPCR validation of expression changes detected by RNA-Seq.

Figure S4. Non-linear relationships between gene expression and stomatal conductance (*g*_s_)

Table S9. Putative transcription factors associated with physiological traits or metabolite abundances.

| Table S1. Tissue sampling and sequencing depth for RNA-Seq analysis  of drought stress and recovery treatments | | | | |
| --- | --- | --- | --- | --- |
| Sampling  date | Time  of day | Treatment | *n* | Average no.  reads aligned |
| Day 13 | 5:00 AM | Drought | 12 | 2,681,431 |
|  |  | Control | 6 | 1,601,913 |
|  | 2:00 PM | Drought | 15 | 2,163,236 |
|  |  | Control | 7 | 1,245,287 |
| Day 14 | 5:00 AM | Drought | 13 | 4,214,697 |
|  |  | Control | 8 | 8,502,935 |
|  | 10:30 AM | Drought | 7 | 2,504,661 |
|  |  | Recovery | 7 | 2,114,300 |
|  |  | Control | 6 | 2,195,533 |
|  | 12:00 PM | Drought | 6 | 1,555,181 |
|  |  | Recovery | 7 | 1,292,167 |
|  |  | Control | 7 | 1,421,956 |
|  | 2:00 PM | Drought | 6 | 1,283,801 |
|  |  | Recovery | 6 | 1,442,102 |
|  |  | Control | 7 | 1,249,621 |
| Totals |  |  | 119 | 303,871,257 |

| Table S2. Construction of transcriptome reference for RNA-Seq. | |
| --- | --- |
| Reads assembled | 3.2 Gb |
| Assembly length | 65 Mb |
| Average coverage | 49.6× |
| Number of isotigs | 89,850 |
| Average isotig length | 1,538 bp |
| Isotig N50 | 1,865 bp |
| Number of isogroups | 38,586 |
| Isogroups assigned gene names | 28,219 |
| Isogroups assigned GO terms | 13,119 |
| Assembly used publicly available EST data from accession Alamo AP13 (454 data from NCBI’s Sequence Read Archive: accessions SRR187765-SRR187775; Sanger sequences from cDNA libraries CFNU, CFNT, and EXTA; Zhang et al., 2013). Reads were quality filtered, screened for adaptor and vector contamination, and assembled in Roche De Novo Assembler v2.6 with the ‘-cdna’ option. | |

Figure S1. Rarefaction analysis of gene discovery as a function of increasing sequencing depth.


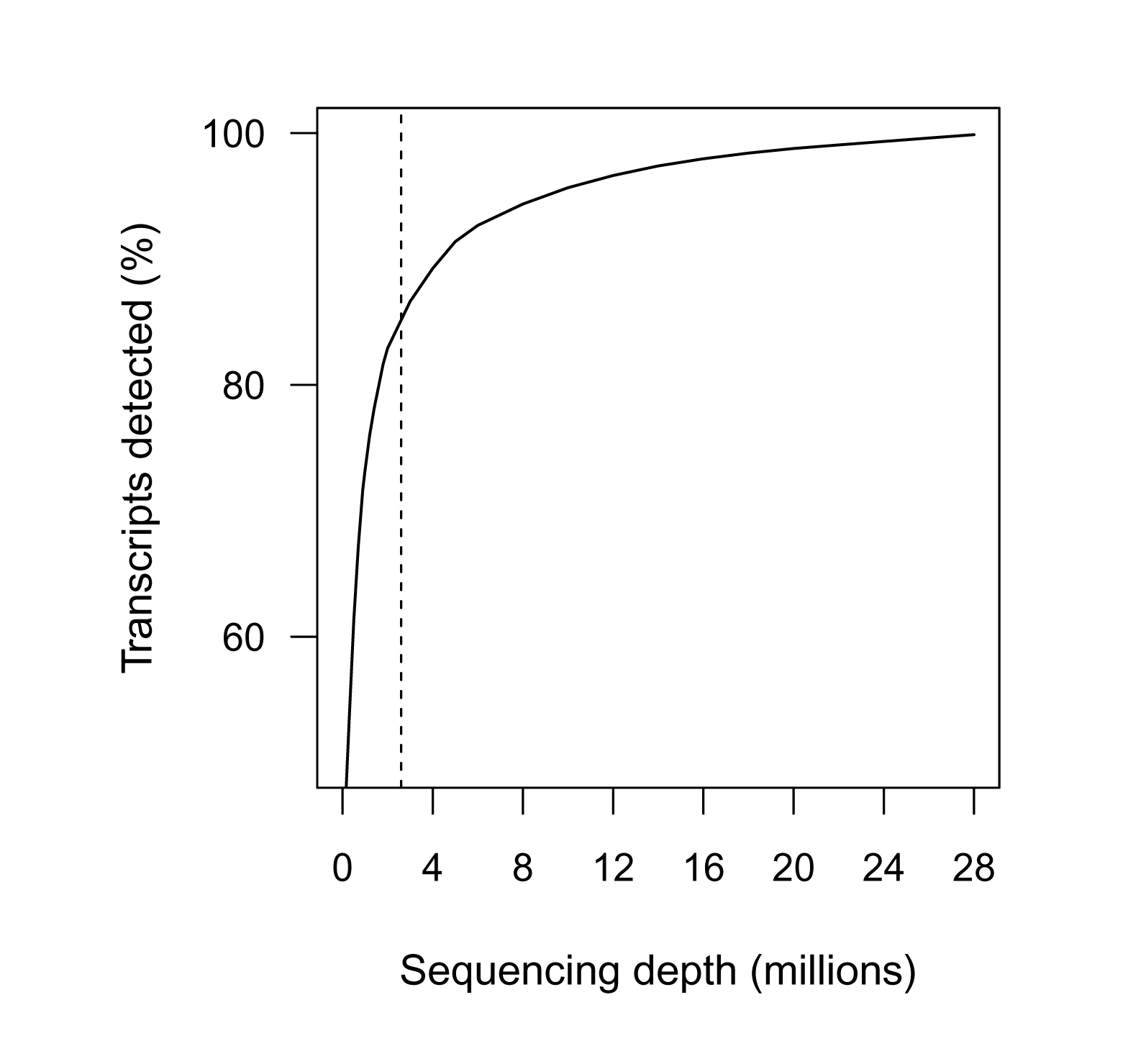


Aligned reads from a single, deeply-sequenced sample were resampled with replacement to simulate a range of different sequencing depths. For each resampled dataset, the number of genes (isogroups) detected at least once was counted. Ten resampled datasets were generated at each simulated depth, and the average number of genes detected per depth is shown here. The vertical dashed line indicates the average sequencing depth per sample achieved in the present study (2.6 million mapped reads per sample), corresponding to 85% of the genes detected at high coverage (>28 million mapped reads).

| Table S4. qPCR targets, primer sequences, and qPCR efficiency data. | | | | | | |
| --- | --- | --- | --- | --- | --- | --- |
| Abbr. | Isotig | Gene Annotation | F primer | R primer | E | R^2^ |
| Ago | 21015 | Protein argonaute 1B (Q7XSA2) | AGTGGGAACATACTGCCTGG | TCTCATCCCACAGGACATGA | 1.89 | 0.99 |
| Alo | 68624 | D-arabinono-1,4-lactone oxidase (Q6YXT5) | TCATCGGAGCAGACACAAAG | GCAAGTTCCTCGAGGTGAAG | 1.81 | 0.98 |
| Arr11 | 40712 | Two-component response  regulator ARR11 (B6UC09) | CTCCTTCCAAGAGCAACCTG | GTGGCATTCTGAGGGTCAAT | 2.01 | 0.91 |
| Ascox | 61335 | L-ascorbate oxidase (B6SZ55) | GTCTTTTCGCGAACTCCTTG | TCGCCAGTCTCCTACTGGAT | 1.91 | 0.82 |
| Cmv | 16587 | CMV 1a interacting protein 1 (B6TUL7) | GGATTTGGAGGAGACGATGA | CCAATCAGTAAGGCACGGTT | 1.85 | 0.98 |
| Cp24 | 29606 | Chlorophyll a-b binding protein CP24 (B4FXB0) | CCTTAACGTCGAGACCCTCA | CATCTTCTACTTCGAGGCCG | 1.94 | 0.97 |
| Dhn | 21267 | Dehydrin (Q41824) | CTTGTGCTCGTGCTTCTCAG | GATCAGGTGGAGGTGAAGGA | 2.01 | 0.93 |
| Dir | 77461 | Dirigent (B6U4X5) | CTCTTACGCAATAGCTCCCG | CACGAAAAGACCCAGGTTGT | 1.84 | 0.96 |
| Gibox | 60093 | Gibberellin 20 oxidase 2 (B6TEH4) | AATGTTGACGAGGAAGGAGC | ACAACTACACGCAGGACACG | 1.95 | 0.96 |
| Grab2 | 59207 | Geminivirus Rep A-binding protein  GRAB2 (Q6Z4N0) | ATTCGTCATCAGACCTTGGC | TTGGGTTCGTGAATCTAGCC | 2.04 | 0.92 |
| Hak9 | 40352 | Probable potassium transporter 9 (Q7XIV8) | CAATATCGCCAGCACTAGCA | CATGGGTCAAGCTGCCTATT | 1.88 | 0.98 |
| Nced1 | 39001 | 9-cis-epoxycarotenoid dioxygenase 1  (Q6YVJ0) | AGGAAGAAGAAGAGCACCCC | CTTGCAGTGCCAATTCTTGA | 1.89 | 0.99 |
| Pp2c | 37128 | Protein phosphatase 2C ABI2 (B6SVM9) | TGCAGCGACAAGTTGTTAGG | TCTGTACCATATGCGTGGGA | 1.92 | 0.97 |
| Prr1 | 29930 | Two-component response  regulator-like PRR1 (B6SVD1) | ACATTGCCAGGAACAAGGAC | GTCCAAAGGTTCAGCAGCTC | 2.13 | 1.00 |
| Snrkip1 | 60623 | SnRK1-interacting protein 1 (B6ST86) | CTAAGAGAGTTGATGGCCCG | GCATGAGCTGGAGCAATACA | 1.94 | 1.00 |
| Usp | 14060 | Universal stress protein (Q7XE49) | GAAACATCTTGATCGCCCAC | CTTACCCCGAGTGCATGATT | 2.09 | 0.84 |
| Zep | 09900 | Zeaxanthin epoxidase (B6U0L0) | TCGGTACCCAACTGTGTCAA | AGGTGATCTTTTGGTCGGTG | 1.97 | 0.95 |
| Eif5a | 03051 | Eukaryotic translation initiation factor 5A  (B6SIL7) | GGTTTTATGCCCTCTGCGTA | CACATCCGTGAAACTGTTGG | 2.00 | 0.98 |
| Cox1 | 67591 | Cytochrome c oxidase subunit 1 (Q5PY52) | TTCTATGGGAGCCGTTTTTG | CCCTAAGAAATGCATGGGAA | 2.07 | 0.99 |
| Cyct1-3 | 29381 | Cyclin-T1-3 (Q2RAC5) | TGAAGCCTCATTCCCAAATC | CGGCAAACTTGGTAATTCGT | 1.86 | 0.95 |

Figure S2. Stability of qPCR reference genes


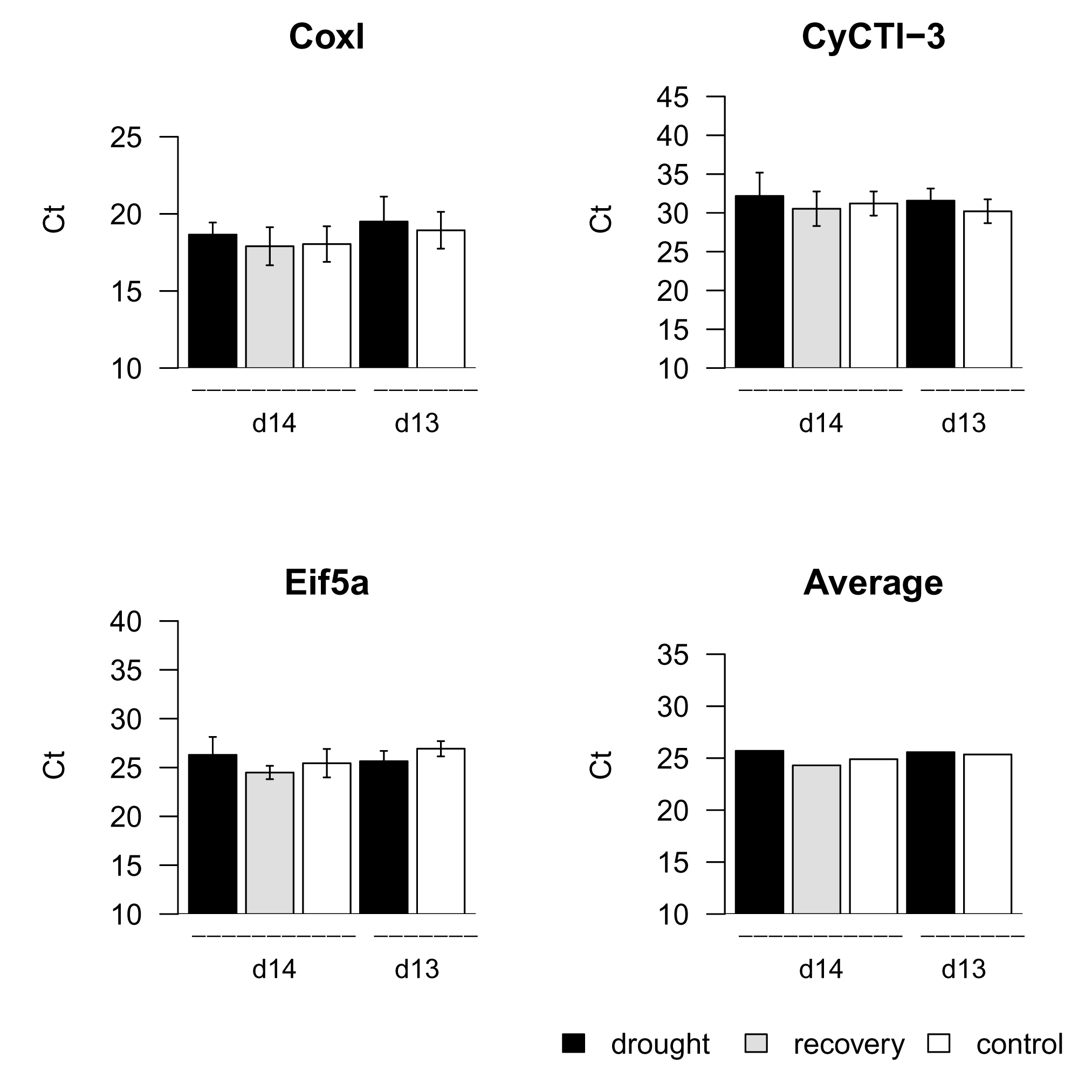


To verify stable expression across samples and treatments for reference genes chosen from RNA-Seq, cDNA was prepared from equivalent amounts of total RNA for all samples (500 ng). Each bar represents the average of n=4 replicate samples from each day and treatment, with each sample assayed in triplicate. Error bars indicate standard error. No differences among treatments or days were observed (ANOVA; P > 0.05), confirming the suitability of these reference genes for expression analysis.

Figure S3. Gene-by-gene qPCR validation figure


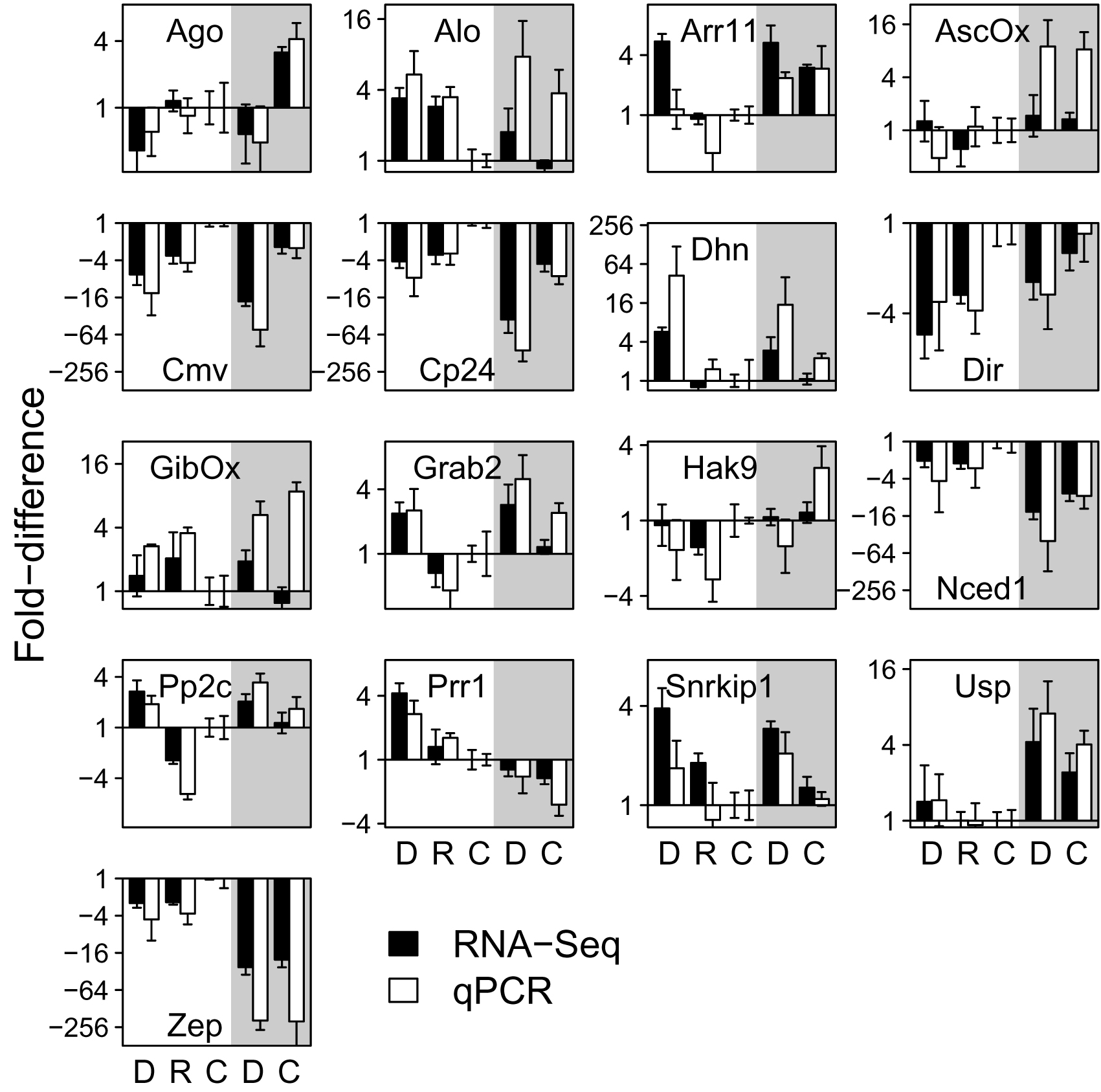


Details of gene expression data for the regression between qPCR and RNA-Seq shown in Figure 5. Each bar represents the average of n=4 samples chosen for validation. qPCR values were measured in triplicate for each sample. Error bars represent standard error of the mean across biological replicates. qPCR data normalized using internal reference genes CoxI, CyCTI-3, and Eif5a. D: drought treatment; R: recovery; C: control.

Figure S4. MIC curves for gs


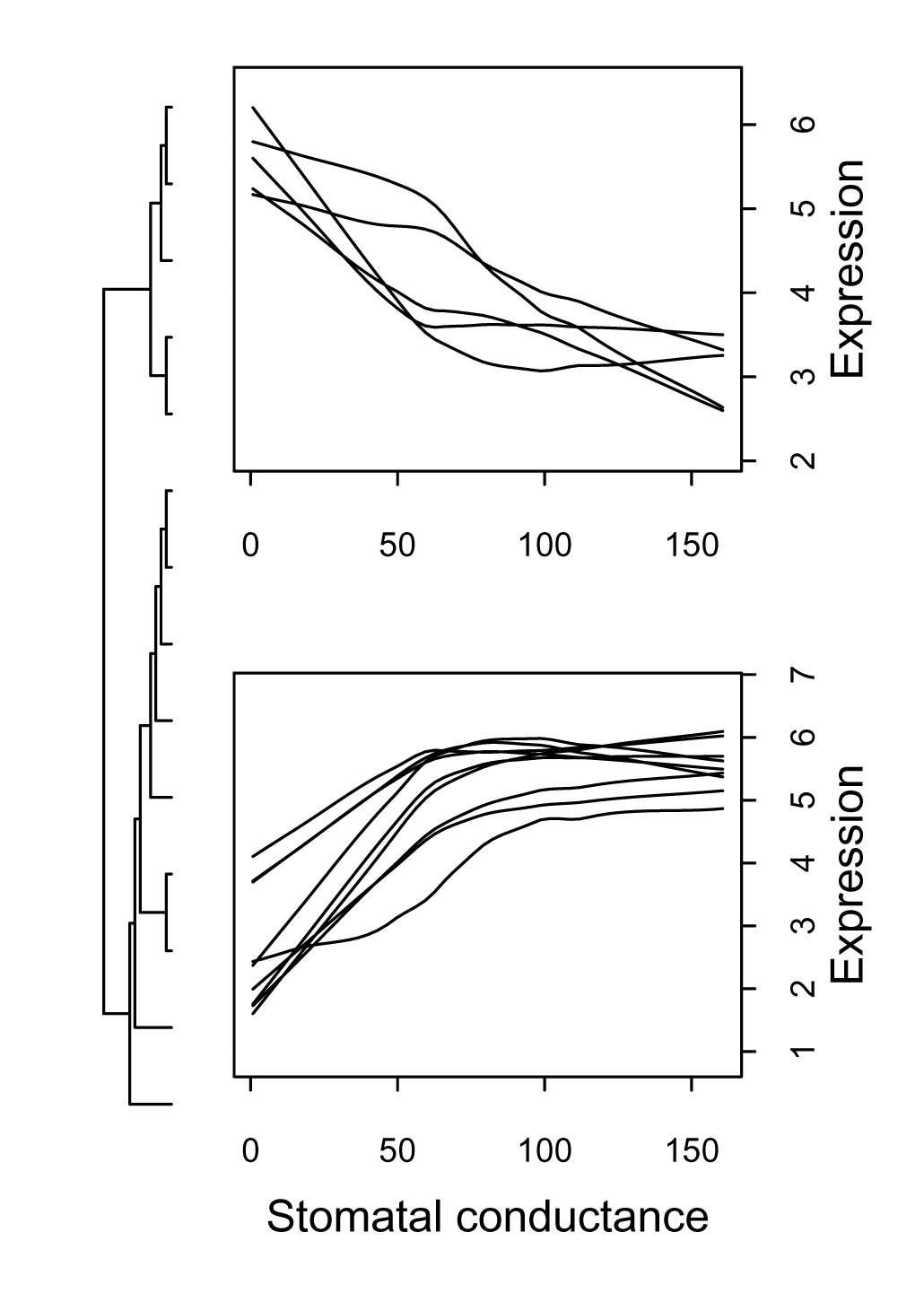


Significant relationships between gene expression and stomatal conductance (*g*_s_) (MIC P < 0.05 after Bonferroni correction, |r| < 0.8). Transcripts showing similar patterns were grouped by hierarchical clustering of dissimilarity matrices. Each line represents a series of paired expression data and physiological measurements smoothed using local polynomial regression (span=0.8).

| Table S9. Putative transcription factors associated with physiology or metabolites | | |
| --- | --- | --- |
| Isogroup | Gene annotations (BLASTX matches) | Correlated trait (*r*) |
| 07796 | Nuclear transcription factor Y subunit B-4 (Q65XK1) | capric acid (0.928) |
| 22061 | BZIP transcription factor protein (B6SNH0) | erythronic acid (0.907) |
| 01272 | Scarecrow (A5HJS4) | erythronic acid lactone (0.928) |
| 13243 | Transcription factor HY5 (B6UHC7) | homoserine (-0.927) |
| 00213 | WRKY transcription factor 18-like protein, methyltransferase-like (Q5QMC1,Q8S0I2) | isocitric lactone (-0.909) |
| 00687 | Steroid hormone receptor TF (D7L9T8) | MPa (0.646) |
| 02136 | Homeodomain protein JUBEL1 (B6SXN6) | MPa (0.719) |
| 01699 | Homeobox-leucine zipper protein HOX29 (A2WLR5) | MPa (-0.72) |
| 03539 | Ocs element-binding factor 1 (B4FB01) | MPa (0.727) |
| 12779 | Homeobox-leucine zipper protein HOX22 (Q7XUJ5) | MPa (-0.729) |
| 12088 | Putative NAC transcription factor (D7R5Z9) | MPa (-0.757) |
| 01380 | Homeodomain leucine zipper CPHB-5 (B6T963) | MPa (0.76) |
| 01815 | Transcription factor AP2D23-like (Q2TQ34) | MPa (0.763) |
| 03044 | DNA binding protein, Putative I-box binding factor (B6U2E0,Q5N7G4) | MPa (-0.772) |
| 04447 | G-box-binding factor 4 (B6SKU0) | MPa (-0.775) |
| 22199 | Homeobox-leucine zipper protein HOX22 (Q7XUJ5) | MPa (-0.783) |
| 00514 | Hydrolase, Trichomeless2, Myb family transcription factors (B6TAN9, D3GKX6, D7LC50, Q9M157) | MPa (0.786) |
| 05832 | Homeobox-leucine zipper ATHB-6 (B6U539) | MPa (-0.801) |
| 06009 | Myb transcription factor (Q5TKI8) | MPa (-0.821) |
| 07067 | WRKY55-superfamily of TFs having WRKY and zinc finger domains (B6SMI9) | palatinitol (0.919) |
| 03191 | G-box binding factor 1 (Q41735) | qP (-0.674) |
| 19717 | R1MYB1 protein (B9UK63) | qP (-0.703) |
| 08677 | Dehydration responsive element binding (B6SSG7) | qP (-0.802) |
| 04835 | Putative Myb13 protein (Q6YSZ5) | threonic acid (0.908) |
| 13142 | TF jumonji domain, N-acetyltransferase and TF-like (D7KZW7,Q6YVS8) | xylonic acid (0.932) |
